# A Review of Polychlorinated Biphenyls (PCBs) Pollution in the Air: Where and How Much Are We Exposed to?

**DOI:** 10.3390/ijerph192113923

**Published:** 2022-10-26

**Authors:** Naffisah Othman, Zaliha Ismail, Mohamad Ikhsan Selamat, Siti Hamimah Sheikh Abdul Kadir, Nur Amirah Shibraumalisi

**Affiliations:** 1 Department of Public Health Medicine, Faculty of Medicine, Universiti Teknologi MARA Sungai Buloh Campus, Jalan Hospital, Sungai Buloh 47000, Malaysia; 2 Department of Biochemistry, Faculty of Medicine, Universiti Teknologi MARA Sungai Buloh Campus, Jalan Hospital, Sungai Buloh 47000, Malaysia; 3 Department of Primary Care Medicine, Faculty of Medicine, Universiti Teknologi MARA Sungai Buloh Campus, Jalan Hospital, Sungai Buloh 47000, Malaysia

**Keywords:** inhalation, indoor, outdoor, air, non-dietary, polychlorinated biphenyls

## Abstract

Polychlorinated biphenyls (PCBs) were widely used in industrial and commercial applications, until they were banned in the late 1970s as a result of their significant environmental pollution. PCBs in the environment gained scientific interest because of their persistence and the potential threats they pose to humans. Traditionally, human exposure to PCBs was linked to dietary ingestion. Inhalational exposure to these contaminants is often overlooked. This review discusses the occurrence and distribution of PCBs in environmental matrices and their associated health impacts. Severe PCB contamination levels have been reported in e-waste recycling areas. The occurrence of high PCB levels, notably in urban and industrial areas, might result from extensive PCB use and intensive human activity. Furthermore, PCB contamination in the indoor environment is ten-fold higher than outdoors, which may present expose risk for humans through the inhalation of contaminated air or through the ingestion of dust. In such settings, the inhalation route may contribute significantly to PCB exposure. The data on human health effects due to PCB inhalation are scarce. More epidemiological studies should be performed to investigate the inhalation dose and response mechanism and to evaluate the health risks. Further studies should also evaluate the health impact of prolonged low-concentration PCB exposure.

## 1. Introduction

Polychlorinated biphenyls (PCBs) are a group of toxic environmental pollutants categorized as persistent organic pollutants (POPs). The commercial production of PCBs started in 1929. Their main use was in electrical and hydraulic equipment and construction materials. Because of their significant adverse effects on human well-being and ecosystems, the manufacturing of PCBs was banned in the United States in 1979 under the US Toxic Substance Control Act [1]. Nevertheless, production continued elsewhere. As a result, the Stockholm Convention was established by the United Nations Environment Programme in May 2001, with the aim of eliminating PCB-containing products by 2028 in an environmentally sound manner [2]. Following this effort, the international total ban of PCBs came into effect on 17 May 2004.

There is still a long way to go, as only 3 million tons of PCB products had been eliminated as of 2015. Meanwhile, 80% of PCBs (17 million tons) are still in the environment, highlighting that the risk of exposure has not yet been eliminated [3]. Even with the production of PCBs banned in most countries, they continue to pose a threat due to their environmental persistence and bioaccumulation, raising global concerns. PCBs are still slowly and continuously being released by products that were manufactured before the ban and that are dumped as waste into the environment [4,5]. They are found in the ambient air and the food chain, and can be transmitted to humans through the ingestion of contaminated food products, inhalation, or transdermal exposure. PCBs have been detected in various food samples, and human exposure has been reported in several countries worldwide [6,7,8].

## 2. Polychlorinated Biphenyls (PCBs)

**Figure 1:**
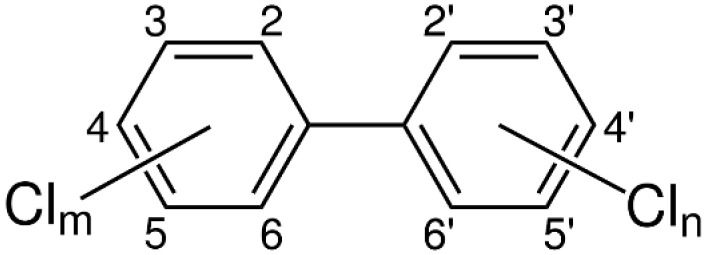
Chemical structure of PCBs (m and n denote number of chlorine atoms on each ring).

Polychlorinated biphenyls (PCBs) are a group of synthetic aromatic chemicals. They consist of a biphenyl structure that is bound to hydrogen and chlorine atoms according to the chemical formula C_12_H_x_Cl_y_, where x and y range from 1 to 10, and x + y = 10. The chemical structure of chlorinated biphenyls is shown in Figure 1. According to IUPAC, there are 209 PCB congeners, which differ by the number of hydrogen atoms substituted by chlorine atoms and their position on the biphenyl rings. This variance in PCB molecule determines the physical and chemical properties and toxicity.

The phenyl rings can rotate around the C-C bond, but with an increasing number of chlorines in the ortho positions (2, 2, 6, 6), this rotation is hampered. With no ortho-chlorines, the two phenyls can align co-planarly [9]. The plane between the phenyls as the C-C bond attain increased double bond characteristics is stabilized by the aromatic system. However, with an increased number of chlorine atoms in the ortho position, the likelihood that planarity will be established decreases. This is significant for the toxicity of the individual congener, as the toxicity of co-planar non-ortho congeners have a “dioxin-like” toxicity [10]. Mono-ortho congeners may also be somewhat co-planar, but if there are two or more chlorines in the ortho position, co-planarity is not possible.

## 3. Why Concern about PCBs

PCBs are among the 12 initial POPs called the “dirty dozen” under the Stockholm Convention [11], listed in Annex A (elimination) and Annex C (unintentional production). They are of global concern due to their (1) persistence in the environment, (2) long-distance travel in the atmosphere, (3) bioaccumulation, and (4) biomagnification in the food chain. It is a global issue, as everyone is likely to have some amount of PCB in their body from either the ingestion of contaminated food, inhalation, or dermal exposure to a polluted environment. Thus, PCBs can significantly impact animal and human health and the environment.

The consumption of contaminated food is often regarded as the major source of human PCB exposure, and there have been limited studies on the role of air pollution as an inhalational pathway. Because of the high PCB concentrations in some animals, dietary exposure has traditionally been prioritized in studies over dermal and inhalation exposure. However, the inhalation route of PCB exposure is often overlooked. A study reported decreased PCB concentrations in food, highlighting that inhalation may be an essential route of exposure [12]. Airborne emissions of PCB may result in inhalation exposure levels comparable to, and occasionally more significant than, dietary ingestion [13].

Atmospheric PCB is a significant contributor to the total body burden, as evidenced by elevated blood levels of lower-chlorinated PCBs (dominant congeners) in the air by 40% among occupants of contaminated buildings [14]. For people living in buildings with significant PCB levels in the building materials, the bulk of their overall PCB burden comes from exposures in their homes, nearly 40 years after the Danish ban on the use of PCBs in construction products [15]. Following exposure, PCB can bioaccumulate and persist in the human body for up to 12 years [16]. Many lower-chlorinated congeners are likewise endocrine-disruptive [17] and carcinogenic [18]. This indicates that inhalation and dermal absorption are significant routes of exposure to PCBs in the air [19]. This review discussed the occurrence of PCBs in the air as a source of inhalation exposure and the subsequent impacts on human health in light of the increased interest in this study area.

## 4. Occurrence of PCBs in the Air

The primary source of PCBs in the atmosphere is the volatilization of PCB-containing products in landfills that are disposed of as waste [20,21]. The widespread use of PCBs in commercial and industrial products and their inappropriate disposal have created severe environmental contamination. Today, PCBs can still be released into the environment from the following sources: (1) poorly maintained hazardous waste sites that contain PCBs; (2) illegal or improper dumping of PCB wastes into landfills that are not designed to handle hazardous waste; (3) accidental spills and leaks during the transport of the chemical; (4) leaks or fires from electrical transformers, capacitors, or other products containing PCBs; and (5) waste incineration and open burning in landfills that emit PCBs during the combustion process [1].

**Figure 2:**
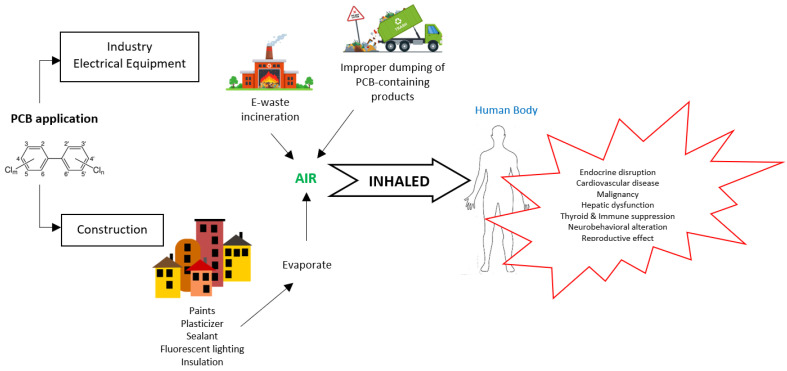
Sources of PCB in the air, human exposure, and possible health impacts.

Once in the environment, PCBs do not readily break down. Instead, they undergo chemical biotransformation that allow them to continue cycling between air, water, and soil for an extended period. PCBs in the air are generally lower-chlorinated (≤5 chlorine atoms), and are mainly taken up by humans via inhalation and dermal resorption. In contrast to high-chlorinated congeners (>5 chlorine atoms), lower chlorinated PCBs exhibit a faster elimination rate from the body and a lower environmental persistence. Figure 2 shows the sources of PCB in the air, human exposure and possible health impacts.

### 4.1. Outdoor Environments

Despite the restricted use of PCBs for nearly four decades, PCB residues have been found in various environmental matrices across the globe. Interestingly, the concentrations of PCBs vary seasonally: they are higher in the soil during winter and higher in the air during summer [22]. This seasonal variation suggests that the emission of PCBs from the soil increases during hot seasons due to volatilization, especially low-chlorinated PCBs. The standards and regulations are designed for the occurrence of PCBs in the environment to protect humans from probable adverse health effects by the U.S. government (Table S1) [23].

#### 4.1.1. E-Waste Recycling Areas

The atmosphere has a significant impact on the long-term transformation and distribution of POPs, including PCBs. The emission sources of PCBs in the environment have seen a shift from intentionally produced PCBs to a combination of intentionally and unintentionally produced and e-waste [24]. In areas where severe PCB pollution in the atmosphere has been reported, burning and recycling of electrical and electronic waste (e-waste) have become a primary source. In the course of recycling e-waste, processes such as manual dismantling, shredding, roasting printed circuit boards, acid-stripping metals, and open burning of e-waste can release these contaminants as unintended by-products [25]. The amount of PCB released to the environment can differ depending on the type of e-waste and the operating technique. High temperatures utilized during the disassembly process can lead to significantly higher PCB releases into the atmosphere. The highest concentration of PCBs were detected in heating furnaces and incinerators in shops working on televisions and hard disks [26].

Similar to other POPs, the peak concentrations of PCBs in the atmosphere can be attributed to emissions from the uncontrolled burning of solid waste, which contaminates surrounding areas [27]. A high concentration of atmospheric PCB has been reported in e-waste disassembling areas in both southern [28] and northern China [29]. In South Asia, the PCB concentration in the surface soil near informal e-waste recycling sites was approximately 23-fold higher than in open dumpsites [30]. Exposure to PCB near e-waste sites is closely related to hormone disruption, especially in children [31]. Furthermore, high PCB concentrations were reported in the blood of e-waste workers following occupational exposure [32,33] and individuals living near e-waste recycling plants [34]. These findings imply that simple e-waste disassembly processes could also be a significant source of PCB emissions.

With effective waste management becoming increasingly challenging, municipal solid waste incineration (MSWI) has emerged as an alternative in developed countries. Even though MSWI is aimed at recovering energy and reducing volume, it has been linked to the emissions of heavy metals and toxic chemicals, along with volatile organic compounds and polyaromatic hydrocarbons, which pose a threat of cancerous and non-cancerous diseases among the nearby population [35,36]. Recent studies have shown that municipal solid waste incinerators are among the most significant contributors to environmental pollutants. The authors of [20] reported decreasing atmospheric PCB levels with increasing the distance from the emission source.

#### 4.1.2. Industrial Areas

PCBs have been widely used in industries over the decades. They can be released through industrial processes such as smelting and cooking, as well as during the burning of coal, wood, crude oil, gasoline, and diesel fuel [37,38]. PCBs in the atmosphere have been detected at higher levels in industrial areas, which are mainly produced unintentionally [39].

Atmospheric PCBs can be found in gaseous or particulate phases. They can bind to particulate matter and be distributed as fine particles due to their low vapor pressure [40]. In Germany, significant gaseous emissions of PCBs were detected from a silicone rubber production site, which was urged to take mitigation measures [41]. In highly industrialized areas, the incidence of chronic illness is likely to grow in parallel with economic growth, with increases in lifestyle diseases and the abundance of carcinogens in the environment [42].

PCBs in the air can be carried long distances and have been reported in regions with no industrial activity, where they have never been used [43]. Consequently, they are found worldwide, although the concentrations might be lower with increased distance from the emission source [20]. The occurrence and homologous patterns of PCBs in the environment can vary depending on chemical volatility, ambient temperature, topography, atmospheric transport, and soil organic matter content, which will predict the potential health risks of human exposure.

#### 4.1.3. Urban vs. Rural Area

Because of the source and usage, PCB concentrations in the air are significantly higher in industrial and urban areas than in remote areas [21,44]. According to Ampleman, Martinez [45], the level of PCB exposure via inhalation is relatively higher among urban than rural residents. With more than half of the world’s population living in urban areas, this risk is worrying.

A clear demarcation of the concentration and occurrence of PCBs is seen between rural, urban, and industrial areas. The PCB concentrations in industrial areas are two to five times higher than in rural areas; in Chile’s urban and industrial areas, high-chlorinated congeners predominate [46]. On the other hand, lower-chlorinated PCBs predominates in the ambient air in Bursa Province [47] and Kutahya, a province in Turkey with a power plant [37]. Greater atmospheric PCB levels are typically observed in megalopolises with significant populations, such as in China [48].

In the United States, the PCB concentration in urban schools close to a PCB-contaminated waterway of Lake Michigan was significantly higher than in schools in rural areas. In fact, the concentration was higher in the indoor environment [49]. In remote areas, much lower atmospheric concentrations of PCB have been recorded. Monitoring of the spatial pattern and temporal trends of PCB at 16 background sites in the Tibetan Plateau showed concentrations ranging from 0.10 to 3.90 pg/m^3^ [50]. The concentrations and dominant congener profiles are summarized in Table 1.

### 4.2. Indoor Environments

While the concentrations of PCBs in the environment can be attributable to various sources, the indoor concentrations significantly exceed those in outdoor air [63]. This highlights that PCBs used and released indoors are more persistent and impact human health, as people spend most of their time indoors.

#### 4.2.1. Building Design

The application of PCBs in construction has led to significant indoor air contamination in buildings [64]. PCBs were primarily used in construction materials such as plasticizers, paint, ceiling tiles, insulation, fluorescent lighting, caulk, and roofing from the 1950s to the late 1970s [65,66,67,68].

As a result of their inert property, PCBs are still present in such materials in homes and schools and will remain there for centuries. In Denmark, the concentration of PCBs in houses was seven-fold higher (2330 ng PCB_total_/m^3^) than the lowest action level (300 ng PCB_total_/m^3^) recommended by the Danish Health Authority [69]. This indicates that possible PCB exposure in the indoor environment contributes to significant overall PCB exposure among the Danish population [14]. With lifetime exposure, the health effects might be more significant. Meanwhile, routine air monitoring in a public building in Aachen, Germany, revealed considerable contamination due to prior use of PCBs in elastic joint sealants, particularly lower-chlorinated PCBs [19].

Aside from building construction, the concentration of indoor PCBs are significantly influenced by the ratio of wall and ceiling area to building volume. Additionally, contaminated air can be absorbed by a tertiary product such as a sealant, which will contribute significantly to the total indoor PCB concentration. Indoor PCB levels were found to be lower in residences with a larger total amount of sealant per volume ratio [70]. Additionally, the interior design of a building can determine the indoor PCB concentration. Carpet pads and wood floor finishes can be residential sources of these semi-volatile organic compounds, as they tend to partition to non-mobile household surfaces [71].

Indoor PCBs are semi-volatile and are slowly but continuously released into the air. Therefore, they can evaporate from their sources and subsequently absorb into surfaces, including dust and bioaccumulate in humans via inhalation and non-dietary ingestion [72,73]. PCBs have been found in dust from homes and other indoor environments worldwide. Indoor exposure to PCBs in contaminated buildings can occur via air and dust inhalation and dust ingestion [49,74]. The inhalation of PCBs from contaminated indoor air may lead to significant PCB levels in the blood [75]. Table 2 shows the concentration and dominant congeners in indoor environments in different settings.

#### 4.2.2. Dust Ingestion

In addition to direct human exposure to PCBs through inhalation, airborne PCBs can contaminate indoor dust, which potentially impacts human health via unintentional ingestion. For example, a study conducted among 26 sample pairings in the United States found that PCBs in vacuum-cleaner dust were positively correlated with the levels of PCBs in human serum [91]. Furthermore, Frederiksen, Andersen [74] reported that residents of buildings constructed with PCB-containing materials that had significant dust PCB levels had high serum PCB levels.

While industrial emissions contribute as the major source of PCBs in urban areas, substantial dust pollution in rural houses poses significant exposure to this pollutant [81]. Approximately a quarter of non-dietary PCB exposure occurs through unintentional dust ingestion, especially among vulnerable groups [92]. In children, an increased risk of childhood leukemia has been linked to higher PCB levels in household dust [93]. At the same time, exposure to PCBs among older adults may contribute to a diminished cognitive ability [94].

For those who live in densely populated, developed regions and areas in proximity to industrial sources of PCBs, the exposure risk is higher in outdoor and indoor settings. In addition to direct exposure, indoor PCB contamination is likely influenced by the occurrence of PCBs in the outdoor air [79]. Similarly, the air in houses with backyard e-waste recycling has two to three times the PCB contamination than indoor air in urban areas [87]. Therefore, all sources of airborne PCBs should be considered when assessing PCB concentrations in indoor air [86].

## 5. Health Impact

PCBs pose a health risk to humans through three routes of exposure (oral ingestion, inhalation, and dermal absorption). While dietary consumption represents a major route of exposure in adults, inhalation presents a two times higher carcinogenic risk, especially among children [86]. In 2015, PCBs were classified as a Group 1 carcinogen (carcinogenic to humans) by the International Agency for Research on Cancer, which further highlights the significance of their oncogenic effects, and makes them a major toxicological concern across the globe [95].

Lerro, Jones [18] described a potential association between PBC exposure and thyroid cancer. PCBs are also proven to be a weakly estrogenic organic compound associated with testicular [96], prostate [97], and breast [98] cancer. In fact, PCB exposure can contribute to cancer aggressiveness and metastasis in women with breast cancer, worsening their prognosis [99]. In a population-based case-control study in the United States in 2005, higher chlorinated PCBs were associated with an increased risk of non-Hodgkin’s lymphoma [100]. Additionally, exposure to PCBs is also associated with the development of colorectal cancer [101], which was the second most common cause of cancer death worldwide in 2020 [102]. Overall, the risk of cancer is 20% higher in men with exposure to PCB [103].

Besides the carcinogenic risk, PCB exposure is also linked to various metabolic diseases. Evidence shows that PCB leads to insulin resistance, which in turn increases the risk of metabolic disorders [104,105,106]. Numerous studies have examined the association between plasma PCB levels and cardiovascular disease risk factors such as hypertension, type 2 diabetes, obesity, and dyslipidemia [107,108,109]. Concerning the health of future generations, studies on the association of serum PCB levels of pregnant women and pregnancy outcomes have shown a significant association with the neonatal thyroid hormone status [110,111]. The effects on infants might be due to in utero exposure [110,112] or breastmilk consumption [113,114,115].

## 6. Conclusions

Inhalation is an essential route of PCB exposure in humans. In this review, we considered scientific papers on environmental PCB contamination. In particular, we discussed the occurrence of PCBs in both outdoor and indoor air and related adverse effects on human health. E-waste recycling is the primary source of PCB contamination in the environment. This phenomenon indicates the need for a remediation strategy to protect the ecosystem from the threat of environmental pollutants.

We emphasized that studies in the literature have reported high PCB concentrations in indoor environments (including in air and dust) originating from building materials (furniture, paints, caulking compounds, and sealants), posing a threat to human health. There is limited knowledge on the available mitigation strategies to reduce PCB levels in the air. Common remediation methods for PCBs in building materials, such as source removal, chemical treatment, and encapsulation of the source, may redistribute the compounds to secondary sources (interior surfaces), causing widespread contamination. Therefore, research should focus on strategies that could help in decreasing high air concentrations of PCBs, especially in indoor environments. With effective mitigation strategies, the effort to eliminate these toxic organic compounds can be accelerated.

Further studies should evaluate PCB risks from inhalation exposure and its dose–response relationship. As it might be disregarded, additional research is needed to assess the health effects of sustained low-concentration PCB exposure.

## Figures and Tables

**Table 1:** Concentration of PCBs in outdoor environments around the world.

Setting	Location	Sample/Sampling Area	Concentration	Dominant Congeners	Reference
**Ambient Air**	Patagonia, Argentina	Ambient air at 11 sites	Σ_38_PCBs: 25 pg/m^3^	PCB-18, 31, 28, 95, 99, 149, 118, and 138	[51]
	Arctic Ocean	Snow surface	ΣPCB flux: 14.4 pg/cm^2^ per year	PCB-5, 11, and 52	[52]
	Fildes Peninsula, West Antarctica	Air samples	Σ_19_PCBs: 1.5–29.7 pg/m^3^	PCB-11	[38]
	Dalian, China	Fine particulate matter	PCBs in PM_2.5:_ 0.04–0.65 pg/m^3^	PCB-105, 138, 118, 101, 153 and 183	[53]
	Tainan, Taiwan	Ambient air at 1 industrial, 2 urban, and 1 rural area	Average dry deposition flux of total PCBs: 0.540–1.94 pg WHO-TEQ/m2 per day	-	[54]
	Hangzhou and Yangtze River Delta, China	Agricultural area (A.A.) and eco-industrial park (EIP)	Σ_29_ PCBs:	AA: PCB-189, 170, and -28 in winter and summer EIP: PCB-189, 170 and -28 in winter; PCB-28 in summer	[55]
			AA: 224–482 pg/m^3^ (winter); 135–553 pg/m^3^ (summer) EIP: 233–537 pg/m^3^ (winter) 39.9–254 pg/m^3^(summer)		
	Valencia Region, Spain	Ambient air at 7 monitoring stations	Σdl-PCB:1.18 to 10.00 fg TEQ/m^3^ Industrial area: 2.20 fg TEQ/m^3^ Urban areas: 3.11 fg TEQ/m^3^	-	[56]
**Urban Areas**	Madrid, Spain	68 ambient air samples	Total PCBs: 437 pg/m^3^	PCB-28, 52, 101, 138, 153, and 180	[22]
	Turkey	32 urban and rural sites	Annual average Σ_43_PCBs: 108 ±132 pg/m^3^.	Rural: PCB-104,114, 118, 123, 151, 167, and 203 Urban: PCB-101, 138, 153 and 118	[57]
			Highest mean at urban sites: 403 ± 428 pg/m^3^ Highest mean at rural sites: 217 ± 353 pg/m^3^		
	Naples, Italy	Atmospheric bulk deposition	Deposition flux of Σ_18_PCBs: 0.075–1.22 ng/m^2^/day	PCB-28, 138, 153 and 180	[44]
**Industrial Areas**	Kocaeli, Turkey	Ambient air at 23 sites	Σ_41_PCB: 4152 ± 6072 pg/m^3^	PCB-18, 28, 31 and 33	[42]
	Eastern China	Ambient air around municipal solid waste incinerator	Σ18PCBs: 81 ± 46 pg/m^3^ (summer); 70 ± 13 pg m^3^ (winter)	PCB-28, 52, 101, and 138	[20]
	North Rhine-Westphalia, Germany	Ambient air at silicone rubber production site	Σ_6_PCB: 300–1500 pg/m^3^	PCB 47, 51 and 68	[41]
	Dilovasi region, Turkey	Ambient air at 23 industrial sites	Σ_41_PCB: 4152 ± 6072 pg/m^3^	PCB-28, 18, 31, and 33	[58]
	Pohang, South Korea	Bulk deposition at steel manufacturing plant	Σ_12_PCB deposition fluxes: 1.3–4.7 ng/m^2^/day	PCB- 77, 118, and 105	[59]
	Aliaga region, Turkey	Ambient air at 40 industrial sites	Σ_35_PCB: 349–94,363 pg/m^3^	PCB-18, 28, 31, 33, 52, and 49	[60]
**E-waste Recycling Sites**	Taizhou, China	17 ambient air samples	Σ_57_PCB: 37.75–65.83 ng/m^3^	-	[61]
	Chennai, India	Ambient air	3.6–53 ng/g	tetra (4-CB), penta (5-CB) and hexa (6-CB) homologs	[62]
	China	Ambient air	7825–76,330 pg/m^3^	-	[28]

**Table 2:** Concentration of PCBs in indoor environments in different settings.

Settings	Location	Sample/Sampling Area	Concentration	Dominant Congeners	Reference
**Workplace**	Hong Kong, China	Air-conditioner filter dust	Σ_37_PCBs: 46.8–249 ng/g	PCB-77, 194, and 199	[76]
	Abraka and Warri, Nigeria	Indoor dust at electronic repair workshop	Σ_28_ PCB: 96.6–3949 ng/g	Hexa-PCB	[77]
	Durban, South Africa	Dust	Σ_3_PCBs: 235 ng/g	-	[78]
	North-Rhine Westphalia, Germany	Air	Σ_28_PCB: 92–2797 ng/m^3^	PCB-28, 52 and 101	[75]
	France	Air	Σ_19_PCB: 1.75 ± 1.82 ng/m^3^	-	[79]
**Resident**	Vietnam	Settled dust	Σ_209_ PCB: 11–1900 ng/g	-	[80]
	Canada	Air	Σ_7_PCB: 455 pg/m^3^	-	[64]
	Czech Republic	Air and dust	Σ_7_PCB: 467 pg/m^3^ (air); 75.1 ng/g (dust)	-	[64]
	Kopawa, Nepal	Dust	Ʃ_30_PCBs: 9.64–16.5 ng/g	Tetra-PCBs followed by penta, hexa, and hepta-CBs	[81]
	Brno, Czech Republic	Air	Σ_7_PCB: 89 pg/m^3^ (summer); 61 pg/m^3^ (winter)	Tri-tetra, and hepta-hexa PCBs	[82]
	Belgium, Italy, Spain, and Portugal	Air	Σ_7_PCBs: 306 pg/m^3^	PCB-28, 52 and 101	[83]
	Farum, Denmark	Air	Ʃ_24_PCBs: 168–3843 ng m^3^	PCB-28 and 52	[84]
	Bursa, Turkey	Air	Σ40PCBs	Penta-, tetra- and tri-CBs	[63]
			Living rooms: 604 ± 210 pg/m^3^ (summer); 362 ± 167 pg/m^3^ (autumn) Kitchens: 639 ± 2514 pg/m^3^ (summer); 309 ± 93 pg/m^3^ (autumn)		
	Brondby Strand Parkerne, Denmark	Air, vacuum cleaner dust, and surface wipes	Σ_15_PCB: 2330 ng/m^3^ (air); 12.000 ng/g (dust); 529 ng/wipe (surface wipes)	Tri- and Tetra PCBs	[69]
	Thessaloniki, Greece	Dust	Σ_15_PCBs: 3.04–9.68 ng/g	PCB-52, 28 + 31 and 101	[85]
	Lahore, Pakistan	Dust	∑_35_ PCB: 0.27–152.9 ng/g	Tri- and Tetra PCBs	[86]
	Hai Phong city and Hung Yen province, Vietnam	Air and Dust	PCBs level in dust: 3.6–320 ng/g PCBs level in air: 1000–1800 pg/m^3^	In dust: penta- and hexaCBs In air: triCBs	[87]
**School**	United States	Air	100–276 ng/m^3^	-	[88]
	United States	Air	0.5–194 ng/m^3^		[49]
	West Midlands, U.K.	Dust	Σ_8_PCBs: 1.2–560 ng/g		[89]
	Indiana and Iowa, United States		0.5–194 ng/m^3^	-	[49]
	Iowa, United States	Air	1.54–35.75 ng/m^3^	-	[90]

## Data Availability

Not applicable.

## Supplementary Materials

The following supporting information can be downloaded at: https://www.mdpi.com/article/10.3390/ijerph192113923/s1, Table S1: Standards and regulations for environmental standards of PCBs.
