# Supplementary material for: A Review of Polychlorinated Biphenyls (PCBs) Pollution in the Air: Where and How Much Are We Exposed to?
Source: Int J Environ Res Public Health. 2022 Oct 26;19(21):13923. doi: 10.3390/ijerph192113923 (PMC9657815; doi:10.3390/ijerph192113923)
Supplement: Supplementary file 1 [file ijerph-19-13923-s001.zip › ijerph-1942301-supplementary.pdf]

International Journal of
Environmental Research and Public Health

MDPI

Table S1. Standards and regulations for environmental standards of PCBs.

Agency	Focus	Level
OSHA	Air (workplace)	1.0 mg/m^3 for 42% chlorinated PCBs<br>0.5 mg/m^3 for 54% chlorinated PCBs<br>For 8 h 5 days per week
NIOSH	Air (workplace)	1.0 µg/m3 for 10 h workday, 40-h workweek.
EPA	Drinking water (environment)	0.0005 ppm
FDA	Food (environment)	0.2–3.0 ppm (all foods)<br>2.0 ppm (fish)<br>10 ppm (paper food-packaging materials)
WHO, FAO	Food (environment)	6.0µg/kg per day (allowable daily intake)
